# A comparative approach for characterizing the relationship among morphology, range-of-motion and locomotor behaviour in the primate shoulder

**DOI:** 10.1098/rspb.2023.1446

**Published:** 2023-10-18

**Authors:** Erin C. S. Lee, Nathan M. Young, Michael J. Rainbow

**Affiliations:** ^1^ Department of Mechanical and Materials Engineering, Queen's University, Kingston, Ontario, Canada K7L 2V9; ^2^ Department of Orthopaedic Surgery, University of California, San Francisco, CA 94110, USA

**Keywords:** range-of-motion, shoulder, functional morphology, primate locomotion, *Australopithecus sediba*, modelling

## Abstract

Shoulder shape directly impacts forelimb function by contributing to glenohumeral (GH) range-of-motion (ROM). However, identifying traits that contribute most to ROM and visualizing how they do so remains challenging, ultimately limiting our ability to reconstruct function and behaviour in fossil species. To address these limitations, we developed an *in silico* proximity-driven model to simulate and visualize three-dimensional (3D) GH rotations in living primate species with diverse locomotor profiles, identify those shapes that are most predictive of ROM using geometric morphometrics, and apply subsequent insights to interpret function and behaviour in the fossil hominin *Australopithecus sediba*. We found that ROM metrics that incorporated 3D rotations best discriminated locomotor groups, and the magnitude of ROM (mobility) was decoupled from the anatomical location of ROM (e.g. high abduction versus low abduction). Morphological traits that enhanced mobility were decoupled from those that enabled overhead positions, and all non-human apes possessed the latter but not necessarily the former. Model simulation in *A. sediba* predicted high mobility and a ROM centred at lower abduction levels than in living apes but higher than in modern humans. Together these results identify novel form-to-function relationships in the shoulder and enhance visualization tools to reconstruct past function and behaviour.

## 1. Introduction

The shoulder is a complex anatomical region that serves as the principal interface between muscles and tendons connecting the trunk, pectoral girdle and forelimb. It, therefore, plays a central role in facilitating motions of the upper body and extremity. Comparative studies across tetrapods indicate that anatomical variation in the pectoral girdle and proximal humerus plays a significant role in forelimb function by enabling or constraining range-of-motion (ROM) at the glenohumeral (GH) joint [1–4]. Primates, with their wide range of both shoulder morphology and upper-extremity mobility, are a case study in form-to-function relationships [5–7]. Notably, the primate shoulder is capable of circumducting the long axis of the humerus to positions on an imaginary ‘globe' surrounding the joint (through a combination of abduction and plane angle rotation) while also ‘twisting' the humerus about its long axis (through axial rotation). Variations in this basic design are thought to have large effects on mobility reflected in locomotor repertoires spanning the baboon's committed terrestrial quadrupedalism to the gibbon's highly arboreal brachiation. Yet, the association among traits, functional impacts and behavioural outcomes remains correlational.

A variety of traits and associated linear measures, ratios, and angles have been proposed to capture these inferred form-to-function relationships in primates. For example, traits possessed by species that engage in frequent overhead postures (e.g. hominoids)—a more globular humeral head with a large articular surface area [5,8,9], a smaller and rounded glenoid [5,10], and a high humeral inter-tuberosity angle [11]—are generally assumed to enable high mobility. On the other hand, traits exhibited by quadrupedal species (e.g. cercopithecids)—a narrow-lipped glenoid and proximally flat humeral head with prominent tubercles that promote stability of the joint through the parasagittal plane [11]—are thought to constrain mobility. That said, these traits and their associated measures have significant limitations that impact interpretation and application. First, it is not possible to visualize ROM directly from linear measures. Therefore, it is unclear what the impact of specific trait values on function and inferred behaviours is. Second, not all traits occur together as a simple package, confounding their interpretation. Thus, some traits may better discriminate observed behavioural differences than others (e.g. arboreal versus terrestrial, suspensory versus quadrupedal), while others may capture functions not strictly associated with GH mobility. The challenge of interpreting these form-to-function relationships becomes even more acute in species that exhibit less specialized behaviours or have mixed or mosaic morphologies. This is particularly true in fossil species. For example, how does one reverse engineer the functional ROM and potential locomotory behaviour of *Australopithecus sediba*, a species that possesses a mosaic of GH features that in turn resembles the orangutan (*Pongo*), chimpanzee (*Pan*) and gorilla (*Gorilla*) [12]? To address this gap, we must characterize both the relationship between morphology and mobility (form–mobility) and between mobility and locomotor behaviour (mobility–function) [13].

Previous attempts to directly link anatomical traits and associated measures to primate GH mobility using functional assessments have provided significant insights into these questions, but also reveal limitations in their application. For example, measurements of ROM from dry bones are limited to independent rotations, successively sampling each degree of freedom (e.g. arm raising in the frontal plane) [9,11]. Rotations of cadavers [14], when available, measure passive ROM. However, manipulating the joint with arbitrary force may put the joint into positions that are not necessarily favourable for maintaining stability or transmitting joint forces *in vivo* (see below). Collecting accurate *in vivo* data using imaging technology such as biplanar videoradiography—which yields direct measurement of bone—is in some regards ideal but logistically challenging to capture, and moreover cannot isolate the direct contribution of skeletal morphology to ROM as it includes soft tissue constraints. A critical gap then lies in the link between how one captures relevant traits and derives related functional outcomes such as ROM in order to identify those measures that are more or less important in facilitating behaviour.

More recently, paleontologists have turned to *in silico* simulations to better account for complex joint interactions across all rotational degrees of freedom [15–18]. This approach requires only three-dimensional (3D) bone meshes to predict 3D ROM by systematically manipulating a ‘digital marionette’ through all possible rotational positions, excluding those where bones interpenetrate [2,19–23] or articular surfaces cease to overlap [18,24,25]. Recent computational ROM studies have added translational freedom along a single axis [23] or systematically enabled 3 d.o.f. translations [24,26]. *In silico* approaches that consider all 6 d.o.f. are well suited for investigating primate GH ROM given that GH translations are observed *in vivo* [27,28]. Further, quadrupedal primates exhibit flattened portions of the humeral articular surface [11]; thus, modelling the GH articulation with idealized ball-and-socket geometry would fail to capture the translational–rotational coupling necessitated by the variable articular curvature (e.g. as measured in the human knee [29]). Systematically introducing 3D translations, however, imposes challenges such as determining an acceptable magnitude of sampled translations and interpreting the physiological relevance of ‘translational mobility’ relative to ‘rotational mobility' [24]. Models that optimize translations to maintain articulation at a given rotational pose may address these challenges.

Here, we extend a proximity-driven GH model [30] to simulate all rotational positions while optimizing translations to achieve a target joint proximity between articular surfaces. We then define the skeletal ROM as the collection of positions where the surfaces maintain target proximity, and the bones are free of interpenetration. This approach attempts to limit ROM positions to those where the entire surface of the glenoid can maintain contact with the humeral head. Our assumption that full glenoid contact occurs *in vivo* is based on its contributions to two established mechanisms. First, maximizing the available joint contact area reduces the compressive stress caused by the forces generated from external loading (e.g. weight bearing) and large muscle forces crossing the joint [9]. Second, the primate GH joint has minimal bony constraints and thus relies on passive and active contributions from soft tissue to provide joint stability [10]. In humans, joint stability is partially achieved through the compression of the humeral head into the concave glenoid—a mechanism presumably enabled through contact with the entire curvature [31]. Considering the similarity in basic GH structure across primates, this mechanism can likely be extended beyond humans to other species.

This study introduces a novel approach for quantifying 3D ROM in primates that incorporates both morphological analyses and *in silico* simulation in a comparative framework. First, we use our proximity-driven GH model to quantify 3D ROM. We next combine existing and novel methods to quantify both ROM magnitude [32] and ROM location (e.g. overhead versus lateral) in living primates and assess which metrics are capable of distinguishing between locomotor groups. We then use shape-based geometric morphometrics to identify morphological features that are correlated with GH ROM and test long-standing assumptions regarding features specialized for mobility. Finally, we apply our model to infer the GH ROM functionality of the fossil hominin *A. sediba* and compare these results directly to living primates.

## 2. Methods

### (a) Scapula and humerus bone meshes

We acquired scapula and proximal humerus bone meshes for species spanning phylogeny and diverse locomotor groups (see electronic supplementary material S2). The brachiator group included *Hylobates* (*n* = 2), *Symphalangus* (*n* = 1) and *Ateles (n* = 1). The more general suspensory group included *Pongo* (*n* = 1) and *Pygathrix* (*n* = 1). The knuckle-walking group consisted of *Pan* (*n* = 3) and *Gorilla* (*n* = 2). The bipedal group consisted of modern humans (*Homo sapiens*) acquired from two previous studies (*n* = 22, see electronic supplementary material S2) [30,33]. Slow climbers included *Potto* (*n* = 1) and *Nycticebus* (*n* = 1). *Cebus* (*n* = 1) comprised the arboreal quadruped group, and *Macaca* (*n* = 1) and *Mandrillus* (*n* = 1) comprised the terrestrial quadruped group. We included a dog (*Canis*, *n* = 1) as a quadrupedal outgroup. The fossil *A. sediba* (*n* = 1) was not assigned an *a priori* locomotor group.

**Figure 1.**
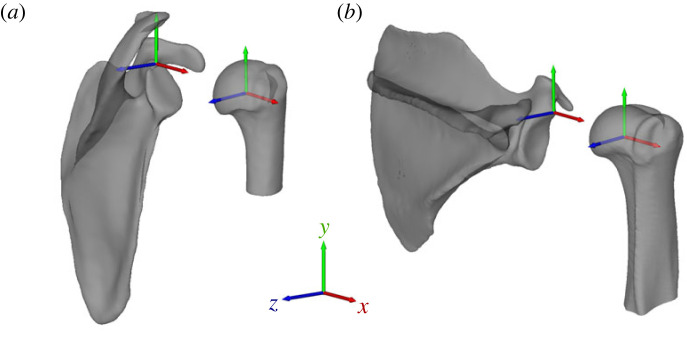
Scapula and humerus anatomical coordinate systems visualized for (*a*) chimpanzee (*Pan troglodytes*) and (*b*) mandrill (*Mandrillus sphinx*).

Several meshes were acquired directly from open-access databases [34], while most meshes were manually segmented in Mimics (Materialise NV, Leuven, Belgium) from open-access computed tomography (CT) images (see electronic supplementary material S2). We smoothed all bone meshes in Geomagic Wrap (3D Systems, Rock Hill, SC, USA). We defined anatomical coordinate systems of the scapula and humerus to maintain consistency across all species (figure 1; see electronic supplementary material S3). Briefly, the scapula coordinate system was defined such that the cranial–caudal (*y*) axis was aligned with the medial (vertebral) border, the medial–lateral (*x*) axis was oriented perpendicular to the medial border and in the plane of the scapula blade and the anterior-posterior (*z*) axis was perpendicular to the blade of the scapula. The humerus coordinate system was defined based on proximal morphology, since the distal humerus was truncated in most CT scans. The cranial–caudal (*y*) axis was aligned with the long axis of the proximal shaft, and the location of the lesser tubercle relative to the centre of the humeral head defined the medial–lateral (*x*) and anterior–posterior axes (*z*).

Following the methodology of Marai *et al*. [35], we built distance fields for each subject's scapula, humerus and humeral head articular surface in MATLAB (Mathworks, Natick, MA, USA; functions provided in supporting dataset). For each mesh, 200 × 200 × 200 grid points were distributed on a rectangular prism 50% larger than the mesh's bounding box. Each grid point was assigned a proximity value indicating the shortest distance to the surface of the mesh. The proximity value is positive if it is outside of the mesh, zero at the surface of the mesh and negative inside the mesh. Proximity values can then be interpolated between these points to estimate the distance to the mesh surface from any point located inside the rectangular prism (e.g. any vertex on an articulating mesh).

### (b) Proximity-driven range-of-motion simulation

For each specimen, we simulated 197 173 rotational positions sampling the entire range of plane of elevation angle (−180° to 180°), abduction (0° to 180°) and axial rotation (−180° to 180°) at 5° increments. At each pose, the rotational position was fixed while the humerus was translated to minimize the difference between (i) the simulated joint proximity and (ii) a target joint proximity (see below), with the added constraint that the bones could not interpenetrate. The joint proximity is defined as the mean proximity between the glenoid vertices and humerus articular surface and is distinct from previous measures of *joint spacing* [36] defined by the minimum proximity between joint surfaces. Targeting the mean joint proximity is a simple yet effective approach for ensuring that the humeral head maintains good coverage of the glenoid, since the mean is sensitive to outliers (e.g. regions on the glenoid far away from the humeral head). We used the MATLAB's *fmincon* function to optimize the humerus translations and included a nonlinear constraint preventing the scapula and humerus from interpenetrating. We considered poses to be within the ROM with a 5% proximity threshold (i.e. if the joint proximity achieved by the optimization was within 5% of the target value, see electronic supplementary material, movie S1). We chose a 5% threshold following a sensitivity analysis that found that altering the threshold from 2 to 8% had a marginal effect on the number of poses in the ROM and the resulting ROM metrics (see electronic supplementary material S5). To test our assumption regarding glenoid coverage and examine how predicted ROM compares with *in vivo* ROM, we compared our *in silico* ROM estimates with *in vivo* kinematics measured from biplanar videoradiography in 20 human participants (see electronic supplementary material S11).

### (c) Custom scaling law for estimating joint proximity

The proximity-driven ROM simulation required subject-specific estimates of target joint proximity. Given that the CT scans yield bone meshes that exclude cartilage, the joint proximity between bone meshes should—anatomically—represent the summed cartilage thickness of the humeral head and glenoid and any synovial fluid-filled space between the surfaces. Several cadaveric studies have reported a strong correlation between body mass and cartilage thickness in compressively loaded joints [37–39]; however, they have primarily investigated the knee, and cartilage scaling of the shoulder may differ. One study reported the relationship among shoulder cartilage thickness, cartilage area and body mass across mice, rats, dogs, sheep and cows [40]. However, cartilage thickness in the shoulders of non-quadrupedal primates may not obey the same scaling law given differences in joint loading, and many subjects lack body mass information (including *A. sediba*).

**Figure 2.**
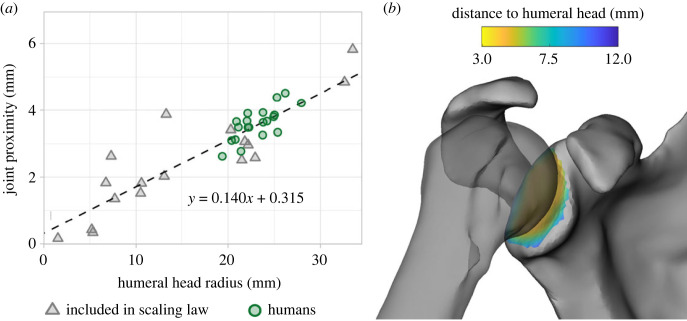
Estimation of joint proximity used as target for simulation. (*a*) Scaling law used for joint proximity estimation, where joint proximity is linearly proportional to humeral head radius. The joint proximity is the mean distance from each glenoid point to the humeral head articular surface in anatomical CT scans, and the humeral head radius is the radius of a sphere fit to the articular surface. Linear regression was performed for subjects (*n* = 17, grey triangles) with anatomical CT scans. Omitted human subjects (*n* = 20, green circles) obey a similar trend. (*b*) Heat map indicating distances from glenoid to humeral head for a Western gorilla (*Gorilla gorilla*) in an anatomical CT pose, with a mean distance (joint space) of 5.8 mm.

We, therefore, developed a custom scaling law that would require only dimensions from scapula and humerus meshes. For each subject with a CT scan acquired in an anatomical joint position, we calculated the joint proximity between the glenoid and humeral head articular surface (figure 2). We then regressed the joint proximity on the radius of a sphere fit to the humeral head articular surface. To establish a relationship spanning primates and non-quadrupedal mammals not skewed by a large sample of a single specie (i.e. humans), we performed the regression on a representative sample (*n* = 17; see electronic supplementary material S4). Joint proximity was strongly correlated with humeral head radius across species (figure 2; *R*^2^ = 0.772, *p* < 0.00001) and, additionally, aligned with the intra-specific trend in joint proximity observed in the current human sample. For the ROM simulations, we calculated the humeral head sphere-fit radius for all subjects and set each subject's target joint proximity according to the scaling law.

We performed a sensitivity analysis to determine how ROM metrics were affected by changes in joint proximity by varying joint proximity target values according to three physiologically plausible scaling laws (see electronic supplementary material S5).

### (d) Quantifying range-of-motion

To quantify the magnitude and location of ROM, we plotted rotational positions according to an adapted version of the spherical rotation coordinate system [41] (see electronic supplementary material S10). In this system, 3 d.o.f. rotations are described in two steps: (i) a long-axis rotation describing the orientation of the humerus long axis on a joint sphere and (ii) an axial rotation describing the rotation of the humerus around its long axis. This spherical rotation coordinate system is advantageous over Euler decomposition as it is sequence-independent and avoids distortion due to gimbal lock. We describe the spherical long-axis rotation by the plane of elevation angle (analogous to longitude) and abduction angle (analogous to latitude). We projected the spherical rotation onto a two-dimensional (2D) map (similar to the projection of Earth onto a political map) using a sine-correction, which ensures that distances between points on the joint sphere are undistorted [32].

**Figure 3.**
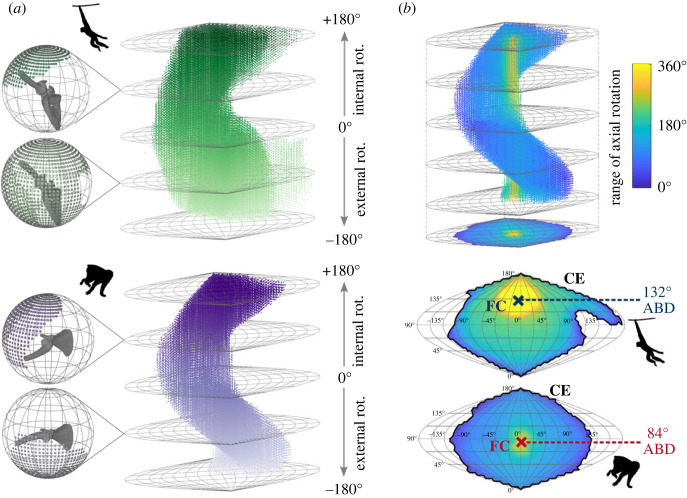
Range-of-motion metrics visualized for the most mobile individual (a lar gibbon; *Hylobates lar*) and the least mobile individual (Japanese macaque; *Macaca fuscata*) in the primate sample. (*a*) ROM at each degree of long-axis rotation is visualized on the glenohumeral joint sphere (see electronic supplementary material, movie S1) and then each joint sphere is mapped onto a 2D projection [32]. The ROM positions are then visualized as a 3D point cloud in angle-angle-angle space. Mobility (the magnitude of ROM) is computed as the volume of the 3D point cloud. (*b*) (Top) The point cloud is projected onto the 2D joint sphere, where each point is coloured according to the range of axial rotation allowed in that pose. (Bottom) The functional centre (FC) locates the point around which rotational freedom is maximized. Only the abduction level of the functional centre (ABD) was analysed. The circumduction envelope (CE, in black) denotes the area on the joint sphere that can be achieved when axial rotation is allowed to vary freely.

We calculated three metrics to summarize ROM (figure 3). First, we computed *mobility*—a measure of the magnitude of ROM—by calculating the volume of an alpha shape encompassing the point cloud in sine-corrected 3D angle space [32] (MATLAB, Mathworks, Natick). We chose an alpha radius of 5 following a sensitivity analysis determining the radius below which the estimated volume significantly decreases (see electronic supplementary material S5). Second, we calculated the *functional centre*—a measure of the location of ROM. We assigned each position on the joint sphere an intensity value according to the range of achievable axial rotation. We then visualized the ROM of each subject as a 2D heat map indicating the degree of axial mobility at each joint pose. We generated a search circle with a radius proportional to the cube root of mobility, within which an average intensity value could be computed. Using MATLAB's built-in genetic algorithm *ga*, we optimized the search circle location to maximize the average intensity value of points within the search circle. The location of the centre of the search circle (the functional centre) is described by its abduction angle and plane of elevation angle. We analysed only the abduction level of the functional centre due to its association with locomotor and postural behaviours. Based on the defined scapula and humerus coordinate systems, this abduction level represents the inclination of the long axis of the humerus relative to the medial border of the scapula. Finally, we computed *circumduction envelope*—a measure of rotational freedom around the joint globe when axial rotation is freely enabled. We calculated the circumduction envelope as the area of an alpha shape encompassing the 2D point cloud of ROM poses projected onto the joint map with an alpha radius of 5. Given the small sample size of each locomotor group, we reported group differences in mobility, abduction level of the functional centre and circumduction envelope by visually comparing distributions rather than through statistical tests. We performed independent linear regressions among the three ROM metrics, performing an *F*-test for a non-zero slope. All statistical tests were performed at a 5% level of significance. To visualize differences in ROM metrics across primate phylogeny, we mapped ROM metrics onto a consensus tree generated from 10kTrees [42] using R packages *phytools* [43] and *ape* [44].

To evaluate the ability of each ROM metric to distinguish function, we first tested whether each ROM metric captured expected differences between the two locomotor groups with the most distinct shoulder function: the brachiators and terrestrial quadrupeds. We also assessed which metrics separated primates from the cursorial outgroup (dog). For subsequent morphological analyses (see below) and for applying insights to *A. sediba*, we retained only ROM metrics that properly distinguished between groups.

### (e) Discrete measures

We tested the relationship between morphology and ROM on a subset of the sample including only hominoids, lorisids and arboreal monkeys. We excluded the terrestrial quadrupeds and the dog from morphological analyses to prevent them from driving trends. Given the large sample size of humans, we included only three representative individuals that possessed the minimum, median and maximum mobilities.

We measured 12 discrete morphological parameters cited as having implications for GH ROM. Angles and linear measurements were calculated from landmarks manually identified in Landmark Editor 3.5 (University of California, Davis). Parameters relating to surface area were computed from areas isolated in Geomagic Wrap. Where appropriate, measures were scaled to scapula centroid size to ensure that shape factors affecting ROM were not skewed by differences in overall body size related to locomotor groups (i.e. large body size of African apes). Further details on the calculation and scaling of each parameter are given in the electronic supplementary material S9. We performed independent linear regression analyses of each ROM metric on each discrete measure, performing an *F*-test for a non-zero slope.

### (f) Three-dimensional geometric morphometrics

Three-dimensional landmark coordinate data (*x*, *y*, *z*) were manually identified in Landmark Editor 3.5 (University of California, Davis). We applied 22 landmarks to the scapula [30,45] and 21 landmarks and 4 semilandmarks to the humerus [46,47] (see electronic supplementary material S8). All subsequent analyses used the R package *geomorph* [48]. Treating the scapula and proximal humerus as separate subsets, we performed local Procrustes superimposition to remove the effects of scale, rotation and alignment. We then combined the scapula and humerus Procrustes shape coordinates by concatenating the separate superimpositions into a common coordinate space. We performed independent linear regression of each ROM metric on the first four principal components (PCs) and identified any PCs correlated with ROM. We visualized the shape axes by warping a thin plate spline of the mean specimen from the minimum to maximum PC scores in the sample [49].

## 3. Results

**Figure 4.**
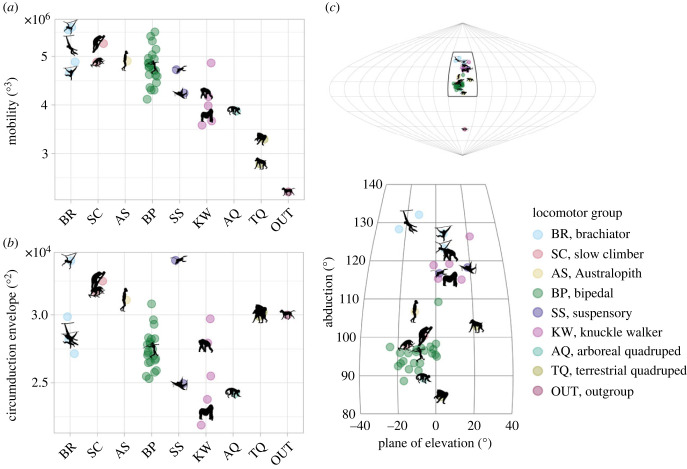
Range-of-motion metrics compared across locomotor groups. Points are individuals (*n* = 40) and silhouettes are species means. Brachiators = lar gibbon (*Hylobates lar*), siamang (*Symphalangus syndactylus*) and spider monkey (*Ateles* sp*.*). Slow climbers = Potto (*Potto* sp.) and slow loris (*Nycticebus* sp.). Australopith = *Australopithecus sediba*. Bipeds = humans (*Homo sapiens*). Suspensory = douc langur (*Pygathrix* sp.) and orangutan (*Pongo*). Knuckle walkers = Western gorilla (*Gorilla gorilla*) and chimpanzee (*Pan troglodytes*). Arboreal quadruped = capuchin monkey (*Cebus* sp*.*). Terrestrial quadrupeds = Japanese macaque (*Macaca fuscata*) and mandrill (*Mandrillus sphinx*). Outgroup = dog (*Canis familiaris*). (*a*) Mobility. (*b*) Circumduction envelope. (*c*) Functional centre, visualized on the 2D map projection of the joint sphere. Top map captures entire joint sphere, including the dog's functional centre. Bottom map is enlarged to see distinctions across primate locomotor groups.

**Figure 5.**
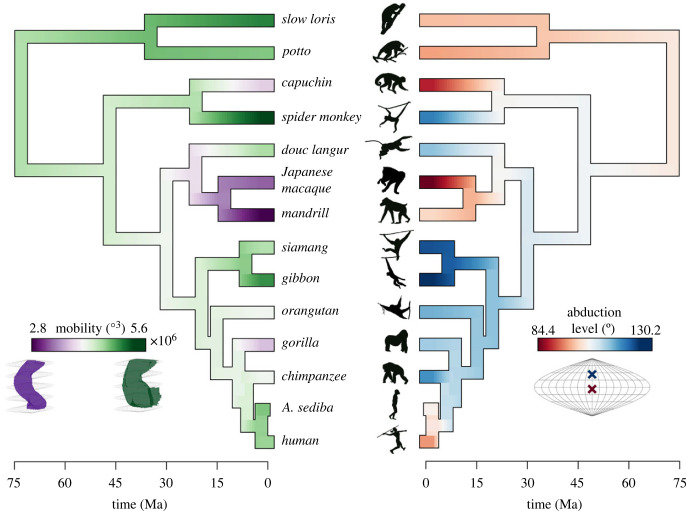
Mobility (left) and abduction level of the functional centre (right) visualized as traits evolved over primate phylogeny. For species with multiple individuals, node colours were determined by the species mean. Non-human apes had consistently high abduction levels, but variable mobility.

We computed all three ROM metrics—mobility, circumduction envelope and functional centre—for 40 individuals representing species spanning locomotor groups (figure 4) and primate phylogeny (figure 5) and a dog for outgroup comparison. Within primates, the abduction level of the functional centre was not correlated with mobility (*R*^2^ = 0.003, *p* = 0.82) or circumduction envelope (*R*^2^ = 0.006, *p* = 0.76), but mobility was strongly correlated with circumduction envelope (*p* < 0.0001, *R*^2^ = 0.635; see electronic supplementary material, figure S14).

Comparing ROM metrics between distinct locomotor groups, brachiators had higher mobility and more highly abducted functional centres than terrestrial quadrupeds (figure 4). Circumduction envelope, however, did not differ between brachiators and terrestrial quadrupeds. Similarly, all primates had enhanced mobility and abducted functional centres relative to the cursorial dog, but the circumduction envelope was not consistently higher in primates than in the dog.

Together, the abduction level of the functional centre and mobility further distinguished intermediate locomotor groups (figure 4; electronic supplementary material, figure S12). Among the higher mobility primates, the brachiator group displayed the most highly abducted functional centres, followed by the suspensory group. The highly mobile slow-climbing lorisids and bipedal humans, however, exhibited lower mean functional centres. Among the less mobile primates, the knuckle-walkers had higher functional centres (with a range overlapping that of brachiators), whereas the quadrupedal monkeys had lower functional centres. *Australopithecus sediba* had mobility consistent with the high-mobility groups (brachiators, bipeds, suspensory and slow-climbers) and a functional centre intermediate to modern humans and non-human apes. When comparing ROM metrics in the context of phylogenetic groups, non-human apes all possessed highly abducted functional centres but variable mobility relative to other primates (figure 5).

Two morphological analyses—conducted on a representative sample of scapulae and proximal humeri (*n* = 18) excluding terrestrial quadrupeds—identified features correlated with ROM measures. Because of their ability to discriminate between locomotor groups, we included only mobility and the abduction level of the functional centre in the morphological analyses. Morphological associations with circumduction envelope are reported in the electronic supplementary material S9. The discrete morphological analysis captured subsets of traits that predicted ROM (table 1, see electronic supplementary material S9). Of the 12 discrete parameters, mobility was associated with features describing a large, spherical humeral articular surface. Functional centre was correlated with features of both the glenoid and proximal humerus; however, the orientation of the glenoid (cranial angle) explained 98% of the variation in the functional centre.

**Table 1.** Results of independent linear regression analyses comparing discrete morphology measures to mobility and the abduction level of the functional centre (see electronic supplementary material S9 for plots). Dashes indicate no correlation, and arrows indicate whether there is a positive (↑) or negative (↓) correlation (*p < 0.05, **p < 0.01, ***p < 0.0001).

morphological parameter	correlation with ROM metric	
	mobility	abduction level
cranial angle	**—**	↑***
critical shoulder angle	**—**	**—**
glenoid surface area	**—**	**—**
glenoid height	**—**	**—**
glenoid width	**—**	**—**
glenoid height : width ratio	**—**	↓*
humerus articular surface area	**—**	**—**
inter-tuberosity angle	**—**	↑**
humeral head radius	**—**	**—**
articular surface area ratio (humerus : glenoid)	↑*	↑*
globularity (humerus radius/centroid size)	↑**	**—**
sphericity of humerus articular surface	↑*	**—**

**Figure 6.**
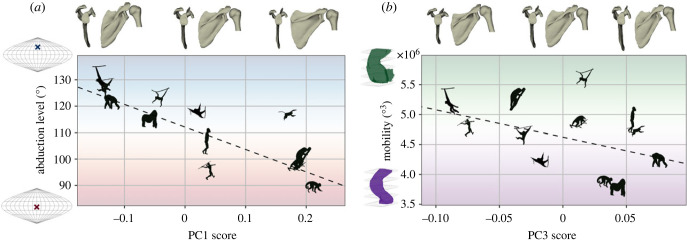
Correlation between ROM metrics and PCs of scapula and proximal humerus shape variation. PCs are visualized by warping the mean shape. Linear regression models were computed from the sample of all individuals in the morphometrics model (*n* = 18; see electronic supplementary material, figures S31 and S33), but species means are depicted here. (*a*) PC1, strongly correlated with functional centre. (*b*) PC3, moderately correlated with mobility.

Three-dimensional geometric morphometrics identified modes of variation that captured the discrete measures above and revealed more complex shape features associated with ROM. The joint-level analysis, including landmarks on the scapula and proximal humerus, produced modes of variation that occurred across the articulating bones (see electronic supplementary material S7). The first four principal components (PCs) accounted for 67% of the total shape variation. PC1 predicted the functional centre (*R*^2^ = 0.587, *p* < 0.001) and explained 30.8% of the shape variation (figure 6; electronic supplementary material, figure S31). A highly abducted functional centre was associated with negative PC1 scores describing a narrow scapula, cranially orientated glenoid, a reduced infraspinous fossa, a rounded glenoid and more retracted humerus tubercles. PC3 was moderately correlated with mobility (*R*^2^ = 0.275, *p* = 0.025) and explained 9.6% of the variation (figure 6; electronic supplementary material, figure S33). Enhanced mobility was correlated with negative PC3 scores describing a broad acromion, a superior-inferiorly short vertebral border, a cranially oriented coracoid process and a large humeral head. Neither PC2 nor PC4 was correlated with ROM metrics.

## 4. Discussion

In this study, we developed a proximity-driven model, and used it to predict the GH ROM of living primate species spanning a range of locomotor behaviours, identified associated shape-based traits and used our results to interpret the fossil hominin *A. sediba*. We found that mobility (the magnitude of ROM) and functional centre (the anatomical location of ROM) are decoupled, meaning high mobility is not necessarily associated with abducted shoulder posture. Our findings suggest that non-human apes are not uniquely adapted for high 3D mobility but are perhaps adapted specifically for overhead ROM as all possess highly abducted functional centres. Morphological analyses revealed that functional centre was correlated with measures cited to be important for overhead behaviour, but mobility was less easily predicted from morphological features.

An important caveat to our bone-based model is that it is most appropriately applied in a comparative framework. We defined the ROM to include any position where the articular surfaces maintained proximity within a defined threshold (essentially modelling glenoid coverage). Indeed, some positions considered acceptable by our model would likely be prevented by soft tissue *in vivo*. For example, in humans, when the humerus is elevated 90°, joint proximity can be maintained for 360° of axial rotation—a motion restricted by ligaments and muscles *in vivo*. Any attempt to predict *in vivo* ROM would require GH soft-tissue properties that are impractical to estimate for all muscles and ligaments across all species and impossible in extinct taxa. We therefore did not intend for our model to replicate *in vivo* ROM perfectly. Comparing simulated ROM with *in vivo* ROM confirmed that, within humans, the proximity-driven model captures the general observation that the humeral head maintains coverage of the glenoid throughout ROM (see electronic supplementary material S11). However, the model does not perfectly replicate *in vivo* translations, and it indeed tends to overestimate *in vivo* ROM. Further testing is required to draw conclusions on the relationship between skeletal ROM and *in vivo* ROM in other primate species. Therefore, our model is more effective for comparing gross differences in skeletal ROM across locomotor groups. Although soft tissue properties will contribute to differences in mobility—and these properties probably vary across taxa—we assume that the bony morphology plays a prominent role in dictating the ROM within which the soft tissue provides further constraints.

To evaluate the model, we tested whether our ROM metrics were consistent with expected differences in ROM of brachiators and terrestrial quadrupeds. Given their clear distinction in locomotor behaviour and morphology, we predicted brachiators would have higher GH mobility, larger circumduction envelopes and abducted functional centres relative to the quadrupeds. We also expected all primates to exhibit higher mobility, higher circumduction envelope and more abducted functional centres than the cursorial dog. We found that mobility and functional region captured the expected distinctions in ROM while circumduction envelope did not (figure 4). Interestingly, relative circumduction envelopes predicted here were similar to the *circumduction area* previously measured in cadaveric experiments, with unexpectedly high circumduction ranges in quadrupeds and low circumduction ranges in brachiating hominoids [14]. This discrepancy suggests that circumduction envelope, or area, is not an effective measure of ROM. While it is logical that circumduction envelope and mobility are correlated—given that both are dependent on the range of positions the humerus can achieve on the joint globe—it appears that the two-dimensionality of the circumduction envelope limits its ability to distinguish groups. We posit that the enhanced ROM possessed by brachiators is elucidated when freedom in axial rotation is incorporated. Given the complex arboreal environment through which *Hylobates* and *Ateles* brachiate, we expect them to require freedom in axial rotation across the joint globe to easily set up their elbow and hand position for gripping substrates overhead [50]. As mobility and functional centre account for differences in axial mobility across the joint globe, they are more suitable for comparing joint ROM and distinguishing function across locomotor groups. This highlights the need for a 3D approach in characterizing primate GH function [51].

Our comparison of ROM across locomotor groups suggests that GH mobility is not specialized for brachiation nor is it unique to hominoids. *Pongo*, *Pan* and *Gorilla* all possessed relatively low mobility while retaining high functional centres. Although all are capable of overhead suspension, they generally move slower and more cautiously through the canopy compared with *Hylobates* and *Ateles*. They perhaps do not require high GH mobility as they can carefully adapt their posture or grip choice to move in a way that their ROM allows. Further, ROM profiles of *Pan* and *Gorilla* suggest that they have lower rotational freedom at lower abduction levels (electronic supplementary material, figure S10). This is perhaps due to their prominent humeral tubercles that presumably enhance the mechanical advantage of the rotator cuff muscles for stabilizing the GH joint during the stance-phase of knuckle walking [52]. *Gorilla* also possessed lower mobility and functional centres than *Pan* (figure 4), which is consistent with their higher degree of terrestrialism [6].

Humans, surprisingly, had mobility on par with brachiating primates but at substantially lower functional centres. Therefore, although the human shoulder is said to be released from arboreal pressures [53], other behaviours important for survival may place a high demand on retaining enhanced mobility at low abduction levels [54,55]. Knapping, tool manipulation and especially throwing all require axial mobility and thus may have shaped the modern GH morphology [56].

While the *A. sediba* scapula has been described morphologically as possessing a mosaic of features resembling *Pongo*, *Pan* and *Gorilla*, its overall GH morphology resulted in a unique ROM profile (see electronic supplementary material S6). Within the context of the hominids, *A. sediba* possesses a functional centre that is intermediate to African apes and humans. This is consistent with its position on the predicted evolutionary trajectory from African apes towards humans in the scapula shape morphospace [57]. This lateralization of ROM combined with *A. sediba*'s high, human-like mobility may reflect a selection for complex tasks at low abduction levels requiring high axial mobility. This is consistent with adaptions at the *A. sediba* hand that are considered to have enabled tool use and precision grip while still enabling a strong grasp for climbing [58,59]. At the GH joint, they retained the ROM to climb or engage in arboreal behaviour to a greater extent than modern humans (consistent with [12,60]), yet they had adaptions that may have allowed *Homo*-like behaviour.

Our morphological analyses suggest that morphological features associated with overhead behaviour are decoupled from features related to enhanced 3D mobility, despite previous assumptions that the two are integrated. For example, select features possessed by brachiating gibbons—a cranially oriented glenoid, a high inter-tuberosity angle and a small rounded glenoid—have been assumed to enable both high abduction levels and high overall joint mobility [10,11,61]. We found that these morphological traits were correlated with highly abducted functional centres but not with overall mobility. Three-dimensional mobility, rather, was correlated with features describing a globular humeral head. PCA further supported our findings, as PC1 and PC3 were correlated with functional centre and mobility, respectively. PC1 and PC3 are orthogonal by definition, indicating that shape changes that affect functional centre (PC1) and shape changes that affect mobility (PC3) can vary independently. This contradicts the notion that mobility is generally linked with frequent overhead arm posture; rather, mobility appears to be primitive for primates in general. Overall, morphological measures best predicted functional centre and not mobility. PC3 accounted for a small amount of shape variation (9.6%), and its correlation with mobility was weak-to-moderate. Our results suggest that morphological features alter 3D mobility in a complex manner, and there are multiple ‘morphological packages' that enhance skeletal mobility. Therefore, for predicting 3D mobility of a new individual or fossil species, simulating ROM may be more suitable than drawing on associations with discrete morphological traits.

A limitation of our study is that it was mainly conducted on open-access bone meshes, and thus we were limited to a small sample size for each non-human species. With our small sample, we observed high intra-specific variability in ROM metrics (particularly mobility) (figure 4). Our sensitivity analyses revealed that the differences in mobility between individuals of the same species cannot be completely attributed to sensitivity in model parameters (see electronic supplementary material S5). Rather, the variability in ROM may be due to intra-specific variation in morphology that has a meaningful impact on function. For example, a large variation in scapula shape across humans alters joint biomechanics in a manner that may explain differences in injury risk [30]. In non-human primates, intra-specific variations in morphology and locomotion are influenced by sexual dimorphism [62,63] and environment (e.g. wild or captive) [64]. Therefore, our sample may be affected by the characteristics of the included individuals. While our inter-specific PC1–PC2 morphospace is similar to that of studies with larger samples [45,57] (see electronic supplementary material S7), we would expect lower numbered PCs to be more sensitive to changes in the sample [65].

The GH ROM presented here does not account for differences in scapular position across species. Further modelling would be required to incorporate scapulothoracic configuration. Variation in where the scapula rests on the thorax alters how we interpret the anatomical meaning of the functional centre relative to the thorax. In hominoids, a long clavicle and a short scapular spine allow the scapula to sit on the dorsal side of the thorax, which orients the glenoid laterally [61,66]. Lorisids, in contrast, have laterally positioned scapulae that orient the glenoid ventrally, thus shifting their GH ROM anterior to the body. Therefore, although hominins and lorisids share similar GH mobilities and functional centres (figure 4), GH abduction manifests more as parasagittal arm-raising in lorisids and frontal plane arm-raising in hominins. A dorsally positioned scapula also enables the upwards rotation of the scapula on the thorax, facilitating higher levels of overall shoulder abduction [67]. The clavicle length of *A. sediba* suggests a dorsally located scapula and a pectoral girdle arrangement intermediate to *Homo* and *Pan*, further supporting a shoulder ROM intermediate to *Homo* and African apes [12].

The scapular morphological characteristics we found here to be relevant for GH ROM could also implicate the scapulothoracic articulation. For example, the mediolateral breadth of the scapula is captured by PC1 (figure 6) and correlates with a more highly abducted functional centre. A narrower scapular blade—represented by low PC1 scores—presumably increases the scapula's range of upwards rotation [68]. While further modelling is required to simulate scapulothoracic ROM, this trait likely further enhances abduction ROM by contributing to scapulothoracic mobility.

The locomotor groups adopted in this study are intentionally broad, as our aim was to understand how summative ROM metrics varied across broad locomotor categories. Indeed, the various species categorized within each group differ in their frequency, technique and kinematics of locomotion. Exploring how these nuanced behaviours correspond with 3D ROM beyond summative metrics would be an interesting avenue of future study. Each individual's 3D ROM has a unique profile with complex 3D interactions, reflecting the extensive variability in ROM not currently captured in our metrics (see ROM projections in electronic supplementary material S6). Future studies focusing on a narrower taxonomic sample are well-suited for exploring the more nuanced variation in 3D ROM and locomotion.

In this study, we used ROM predictions to provide insight into the evolution of the hominoid shoulder and reconstruct the behaviour of the fossil hominin *A. sediba*. We found that enhanced GH mobility is neither unique to brachiators nor common to all hominoids. Rather, interpreting mobility and functional centre as different ‘functions’ enabled a clearer separation of locomotor groups. Further, two morphological analyses revealed that features strongly correlated with high abduction levels were independent of features correlated with mobility. These results suggest that high mobility and adaptation for frequent overhead behaviour are not necessarily coupled. This finding should be considered when evaluating competing hypotheses regarding the evolution of the hominoid upper limb. Considering the similarly high functional centres exhibited across non-human apes, we posit that the common locomotor and postural demand requires ROM spanning high abduction levels, but not high mobility. Kinematic analyses of extant primates engaging in locomotion in the wild, facilitated by recent advances in non-invasive motion capture technology, could provide further estimations of the GH ROM required for various locomotor styles and postures [69].

In conclusion, these results offer a clearer picture of the relationship between GH morphology and ROM such that more informed predictions of ROM of fossil taxa can be made from morphometric analyses alone. Our results demonstrate that the complex interaction of articular features can alter ROM in ways not previously anticipated. Thus, proximity-based simulations, when applied in a comparative framework, can help illuminate function where isolated morphological analyses cannot. While our proximity-driven model was developed for the GH joint, it can be adapted to predict mobility at other joints such as the hip, elbow and knee. Prior to applying the model to other joints, researchers should carefully consider the suitability of proximity-driven translations and identify reasonable joint proximities to target in their ROM simulations. Extending this framework to other anatomical regions and fossil taxa will help to better understand key transformations in primate and hominin evolution.

## Data Availability

Data and scripts required to run the proximity-driven model, compute ROM metrics, and reproduce results presented in the paper are published open-access on the Queen's University Database (https://doi.org/10.5683/SP3/S89LWQ) [70]. The MATLAB functions for computing distance fields developed by J. J. Crisco (Brown University) can be accessed on a public Github repository (https://github.com/BrownBioeng/DistanceFields). Supplementary information is provided in the electronic supplementary material [71].
